# Vertical Etching of Scandium Aluminum Nitride Thin Films Using TMAH Solution

**DOI:** 10.3390/nano13020274

**Published:** 2023-01-09

**Authors:** A. S. M. Zadid Shifat, Isaac Stricklin, Ravi Kiran Chityala, Arjun Aryal, Giovanni Esteves, Aleem Siddiqui, Tito Busani

**Affiliations:** 1 Center for High Technology Materials (CHTM), University of New Mexico, Albuquerque, NM 87131, USA; 2 Optical Science and Engineering (OSE), University of New Mexico, Albuquerque, NM 87131, USA; 3 Electrical and Computer Engineering (ECE), University of New Mexico, Albuquerque, NM 87131, USA; 4 Sandia National Laboratories, Albuquerque, NM 87123, USA

**Keywords:** scandium doped aluminum nitride (ScAlN), wet etching, tetramethyl ammonium hydroxide (TMAH), annealing, thin films

## Abstract

A wide bandgap, an enhanced piezoelectric coefficient, and low dielectric permittivity are some of the outstanding properties that have made ${\mathrm{Sc}}_{x}{\mathrm{Al}}_{1-x}N$ a promising material in numerous MEMS and optoelectronics applications. One of the substantial challenges of fabricating ${\mathrm{Sc}}_{x}{\mathrm{Al}}_{1-x}N$ devices is its difficulty in etching, specifically with higher scandium concentration. In this work, we have developed an experimental approach with high temperature annealing followed by a wet etching process using tetramethyl ammonium hydroxide (TMAH), which maintains etching uniformity across various Sc compositions. The experimental results of etching approximately 730 nm of ${\mathrm{Sc}}_{x}{\mathrm{Al}}_{1-x}N$ (x = 0.125, 0.20, 0.40) thin films show that the etch rate decreases with increasing scandium content. Nevertheless, sidewall verticality of 85°~90° (±0.2°) was maintained for all Sc compositions. Based on these experimental outcomes, it is anticipated that this etching procedure will be advantageous in the fabrication of acoustic, photonic, and piezoelectric devices.

## 1. Introduction

Group III-V materials are getting notable attraction for their diverse applications such as microelectromechanical systems (MEMS), piezoelectric transducers, resonators, and radio frequency (RF) acoustic filter devices [1,2,3,4]. Due to some of its promising qualities and simplicity of process integration, aluminum nitride (AlN) is widely employed in piezoelectric MEMS devices [5,6]. AlN can be doped with other metals to increase its piezoelectric properties [7,8], which advanced the success of ${\mathrm{Sc}}_{x}{\mathrm{Al}}_{1-x}N$ based optoelectronics devices [9,10,11]. At the earlier stage of twenty-first century, Takeuchi et al. used first-principles analysis to determine that wurtzite structure of Sc-IIIA-N alloys can be fabricated [12]. Later, Akiyama et al. demonstrated that by measuring co-sputtered ${\mathrm{Sc}}_{x}{\mathrm{Al}}_{1-x}N$ films [13,14,15] a piezoelectric coefficient of 27.6 pC/N could be achieved and is more than five times higher compared to AlN [13]. Furthermore, according to the experiments of Wingqvist et al. [16], the electromechanical coupling coefficient value of ${\mathrm{Sc}}_{x}{\mathrm{Al}}_{1-x}N$ film can be improved by up to 15%, with recent studies showing coupling coefficients exceeding 20%. Thus, using ScAlN thin films with increasing scandium concentration facilitates the fabrication of high-frequency and wideband acoustic devices [17,18,19]. However, ${\mathrm{Sc}}_{x}{\mathrm{Al}}_{1-x}N$ thin films become challenging to etch as the scandium concentration (x) increases, especially when using reactive ion etching (RIE) or inductively coupled plasma (ICP) etching [20]. Substantial research has been conducted on ${\mathrm{Sc}}_{x}{\mathrm{Al}}_{1-x}N$ to understand its growth and how different etching techniques can be used to fabricate piezoelectric devices [21].

Like any other group III-V material, ${\mathrm{Sc}}_{x}{\mathrm{Al}}_{1-x}N$ can be etched by dry or wet etching techniques. One of the known dry etching approaches is ion beam etching, which can be physical or chemical and can result in smooth etched surfaces at a suitable etch rate [22]. Table 1 summarizes experiments that dry etch ${\mathrm{Sc}}_{x}{\mathrm{Al}}_{1-x}N$ and reports sidewall verticality and etch rate. Luo et al. [23] performed ICP etching with thick S1818 photoresist (PR) as an etch mask. They demonstrated how RF power might control the sample’s plasma etching energy, and how the energy of the ${\mathrm{Cl}}_{2}/{\mathrm{BCl}}_{3}/N_{2}$ plasma enhances the sidewall angle of ${\mathrm{Sc}}_{0.06}{\mathrm{Al}}_{0.94}N$. James et al. demonstrated that the reactive ion beam etching (RIBE) process of ${\mathrm{Sc}}_{x}{\mathrm{Al}}_{1-x}N$ etching is superior to ion beam etching (IBE) in terms of etching rate, selectivity, and sidewall angle (73°) (See Table 1). The ${\mathrm{Sc}}_{x}{\mathrm{Al}}_{1-x}N$ etching rate and selectivity degrade when the identical beam parameters (See Table 1) are used without the reactive gas [24]. Wang et al. [18] presented the design, fabrication, and characterization of ${\mathrm{Sc}}_{0.20}{\mathrm{Al}}_{0.80}N$ thin films used for piezoelectric micromachined ultrasound transducers (PMUTs) by using RIE as an etching process (See Table 1). They claimed that the etched layer has good verticality and concluded that increasing the scandium concentration would enhance PMUT performance [18].

Hardy et al. attempted ICP etching using argon (Ar) rather than the more commonly used nitrogen and found that the etch selectivity is significantly higher for ${\mathrm{Sc}}_{x}{\mathrm{Al}}_{1-x}N$ relative to AlN and the surface roughness can be kept unchanged after etching process [25]. Furthermore, Shao et al. successfully fabricated a lamb wave resonator with a quality factor of around 1000 with ${\mathrm{Sc}}_{0.22}{\mathrm{Al}}_{0.78}N$ by achieving a 77° sidewall [26]. They also concluded that ${\mathrm{Cl}}_{2}$ gas increases the anisotropy of the etching process [26] since the dry etching process is mainly ${\mathrm{Cl}}_{2}/{\mathrm{BCl}}_{3}$ based. However, due to ${\mathrm{ScCl}}_{3}$’s poor volatility, performing dry etching of ${\mathrm{Sc}}_{x}{\mathrm{Al}}_{1-x}N$ can be an ineffective process [20]. On the contrary, it takes more etching power and ion bombardment to get the etching rate back to a considerable level [18,27]. Moreover, it was found that excessive etching into the bottom layer might be a concern for device fabrication, and a lower etching rate as well as high-power consumption are the fundamental causes of poor selectivity to various mask materials, which has accelerated the wet etching trials in the research community [20].

Table 2 summarizes some of the wet etch results of Sc_x_Al_1−x_N reported in literature. The etching rate of Sc_0.15_Al_0.85_N was found to be approximately 50 nm/min (at 60–70 °C) with MIF-319 developer that generally contains 2~5% TMAH [18]. Another work demonstrated that employing 25% KOH at 80 °C, a 500 nm Sc_0.36_Al_0.64_N layer could be etched in only 15 s [28], which indicates KOH as a promising etchant. Several authors highlighted the issues with wet etching lie in the lateral etching behind the mask and the generation of sidewall angle [20,29,30]. Wet etching becomes a highly appealing choice if the lateral etching that occurs throughout the process is not an issue or if it can be minimized to acceptable levels. Another notable observation from Airola et al., is that alkaline etchants will outperform acidic etchants in terms of sidewall roughness and etching rate [20]. They have used different etch masks (Mo/${\mathrm{SiO}}_{2}/{\mathrm{SiN}}_{x})$ with 25% TMAH at 80 °C and concluded that the mask did not make any difference in the etching results [20].

In the recent work of Tang et al. [21], vertical etching of Sc_0.125_Al_0.875_N have been demonstrated by using 10% KOH at the temperature of 65 °C. They successfully etched down 800 nm of ${\mathrm{Sc}}_{0.125}{\mathrm{Al}}_{0.875}N$, but with a significant amount of lateral etching (approximately 395 nm). In addition, the process is not suitable for etching ${\mathrm{Sc}}_{x}{\mathrm{Al}}_{1-x}N$ with higher concentrations as it could not result vertical sidewalls [21]. In this work, we present a wet etching process by using TMAH solution for etching ${\mathrm{Sc}}_{x}{\mathrm{Al}}_{1-x}N$ thin films (x = 0.125, 0.20, 0.40), introducing an intermediary thermal annealing process at 650 °C in a nitrogen atmosphere (The experimental approach and etching results demonstrate a ‘’Universal Method’’ to etch ${\mathrm{Sc}}_{x}{\mathrm{Al}}_{1-x}N$ with sidewall verticality between 85° and 90°.

## 2. Experiment

### 2.1. $Sc_{x}Al_{1-x}N$ Film Deposition

**Figure 1:**
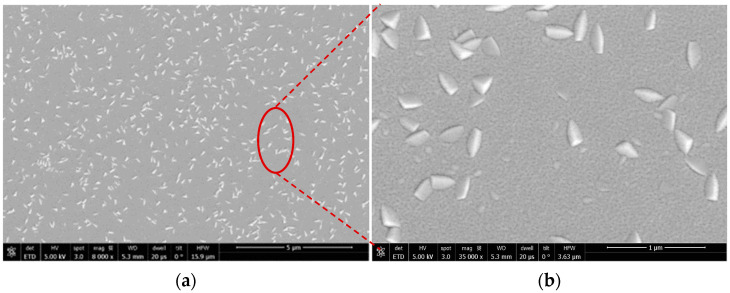
SEM image of the (**a**) ${\mathrm{Sc}}_{0.40}{\mathrm{Al}}_{0.60}N$ surface with abnormally oriented grains (AOG), (**b**) Direction of the AOGs (in micron scale).

An SPTS Sigma 200 deposition system was used to reactively sputter the ${\mathrm{Sc}}_{x}{\mathrm{Al}}_{1-x}N$ films onto 150-mm Si <100> wafers. The conditions for ${\mathrm{Sc}}_{x}{\mathrm{Al}}_{1-x}N$ deposition consist of using 5 kW of target power at 350 °C with a mixture of $\mathrm{Ar}/N_{2}$. Abnormally oriented grains (AOG) nucleate during film deposition and the density varies based on deposition conditions as well as the templating surface. The AOGs increase surface roughness (See Figure 1) and prevents uniform patterning over ${\mathrm{Sc}}_{x}{\mathrm{Al}}_{1-x}N$ for the hard mask needed to define the features to be etched. To quantify the Al/Sc concentration in the films, energy-dispersive spectroscopy (EDS) was conducted, and the Al/Sc ratios fall within the expected values (See Supplementary Materials Sections S1–S4, Figures S1–S24, Tables S1–S18). The Sc_0.80_Al_0.20_N film results were compared to a study by Esteves et al. [31] that used the same sputter target and deposition system to deposit Al_0.80_Sc_0.20_ that had the composition verified by X-ray photoelectron spectroscopy (XPS).

### 2.2. $SiO_{2}$ Film Deposition

**Figure 2:**
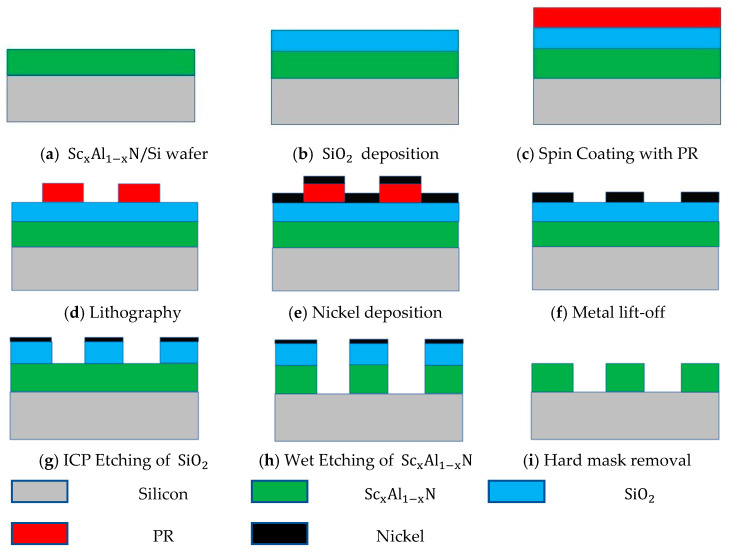
Process flow for sample preparation and etching of ${\mathrm{Sc}}_{x}{\mathrm{Al}}_{1-x}N$ thin films.

A ${\mathrm{SiO}}_{2}$ film was used as a hard mask (See Figure 2) for etching ${\mathrm{Sc}}_{x}{\mathrm{Al}}_{1-x}N$ since ${\mathrm{SiO}}_{2}$ is comparatively much more accessible, well-known in semiconductor processing, and provides good etch selectivity when removing the mask layer post ${\mathrm{Sc}}_{x}{\mathrm{Al}}_{1-x}N$ etch. Before the ${\mathrm{SiO}}_{2}$ deposition, the samples were cleaned with acetone, isopropyl alcohol (IPA), and diluted (DI) water. Though piranha solution (mixture of $H_{2}{\mathrm{SO}}_{4}\;{\mathrm{and}\;H}_{2}O_{2}$) can be used as an alternative cleaning approach [32,33,34], it was found in our experiments that piranha also etches the ${\mathrm{Sc}}_{x}{\mathrm{Al}}_{1-x}N$ film. After cleaning, samples were dried up with a nitrogen gun. The ${\mathrm{SiO}}_{2}$ layer was deposited using a CHA Mark-40 dielectric evaporator. The base chamber pressure reached $1.8\times 10^{-7}$ Torr before deposition and the filament and beam current was maintained at 0.36 A and 6.3~7.5 mA, respectively. The $O_{2}$ backflow (40 sccm) was conducted at the pressure level of $2.5\times 10^{-5}$ Torr. Two different oxide thicknesses of 330 nm and 1 μm were used, and though not shown, the etching quality does not depend upon the mask layer thickness.

### 2.3. Nickel Mask Preparation and Lift-Off

To pattern the ${\mathrm{SiO}}_{2}$ hard mask, a Ni metal film was used as the mask that was patterned using a lift-off process. After ${\mathrm{SiO}}_{2}$ deposition, lithography was conducted using an MLA 150 Advanced Maskless Aligner Heidelberg Instrument. During the process, AZ5214E photoresist (PR) is spin coated onto the sample at 5000 rpm, resulting in a thickness of 1.2~1.4 microns to prepare for a nickel lift-off process for ${\mathrm{SiO}}_{2}$ patterning. The sample(s) were pre-baked at 110 °C for 4 min, then exposed at 405 nm UV light at 135 $\mathrm{mJ}/{\mathrm{cm}}^{2}$, developed in AZMIF-300 for 45~50 s, and rinsed in diluted water for 25–30 s. Subsequently, using e-beam evaporation, a 100-nm thick nickel (Ni) film was deposited, which will eventually serve as a mask during the etching of ${\mathrm{SiO}}_{2}$ layer (See Figure 2). During the Ni-metallization process, the base chamber pressure and evaporation pressure were maintained at $3.3\times 10^{-7}$ Torr, and $1.2\times 10^{-6}$ Torr, respectively. The deposition process was performed at 0.5 Å/s. After Ni deposition, the samples were immersed in an acetone solution for 15 min to remove the lift-off photoresist and expose the required regions for ${\mathrm{SiO}}_{2}$ etching. A post-clean was later used on all samples that consisted of an acetone, IPA, and DI water rinse.

### 2.4. ICP Etching for $SiO_{2}$ Layer

The ${\mathrm{SiO}}_{2}$ layer was etched using an ICP dry etch process after depositing Ni as the hard mask. Initially, the chamber cleaning process was performed for 10 min, where 50 sccm of $O_{2}$ was used with 30 mTorr chamber pressure. The ${\mathrm{SiO}}_{2}$ etch used ${\mathrm{CHF}}_{3}\;\left(45\;\mathrm{sccm}\right)$ and $O_{2}\;\left(5\;\mathrm{sccm}\right)$ gases as the primary etchants during the process, where ICP, RF power, and chamber pressure were, respectively set at 400 W, 100 W, and 5 m Torr. Etch times were based on the ${\mathrm{SiO}}_{2}\;$thickness where a 1 min 50 s etch was used for 330-nm thick layer, and 5 min for 1-μm thick layer. Any residual Ni leftover after ICP process was removed away during the wet etching and HF cleaning process.

### 2.5. High Temperature Annealing

In general, the ions generated by the plasma bombardment and the etched surface transmit kinetic energy during any ICP operation of the ${\mathrm{SiO}}_{2}$ etch. The etched surface can contain a high degree of impurities and defects that originated during the etch process. Wet etching of ${\mathrm{Sc}}_{x}{\mathrm{Al}}_{1-x}N$ immediately after etching the ${\mathrm{SiO}}_{2}$ layer led to a significant amount of surface roughness (See Supplementary Materials Figure S26). Therefore, an intermediate anneal process (Before ${\mathrm{Sc}}_{x}{\mathrm{Al}}_{1-x}N$ etching) was implemented to remove the potential embedded impurities within the ${\mathrm{Sc}}_{x}{\mathrm{Al}}_{1-x}N$ film as well as repair the damaged ions. Airola et al. also demonstrated that high temperature thermal annealing process could be a prospective solution to this challenge [20]. Following the etching of the ${\mathrm{SiO}}_{2}$ layer, we placed our samples in the high-temperature annealing furnace chamber in a nitrogen environment using 40–45 sccm of gas flow and annealed for 1 h at 650 °C. There is a possibility to further induce stress into ${\mathrm{Sc}}_{x}{\mathrm{Al}}_{1-x}N$ during annealing processs, which occurs with the thermal expansion mismatch of the substrate and the ${\mathrm{Sc}}_{x}{\mathrm{Al}}_{1-x}N$ [20]. Therefore, we employed a very modest temperature gradient (20 °C/min) for ramping up the process and cooling down the samples. One of the major concerns about high temperature annealing is that whether it is degrading the film quality or not. Hence, after thermal annealing we checked our sample(s) in SEM and found that there were no significant visual changes. In fact, the AOGs were also clearly visible (See Supplementary Materials Figure S27).

### 2.6. TMAH Wet Etching

The wet etch step used a 25% concentrated TMAH (TMAH: Water in 1:3 ratio) solution at 78 °C~82 °C to etch the ${\mathrm{Sc}}_{x}{\mathrm{Al}}_{1-x}N\;\left(x=12.5\%,\;20\%,\;40\%\right)$ films. The etching rate of ${\mathrm{Sc}}_{0.125}{\mathrm{Al}}_{0.875}N$, ${\mathrm{Sc}}_{0.20}{\mathrm{Al}}_{0.80}N$, and ${\mathrm{Sc}}_{0.40}{\mathrm{Al}}_{0.60}N$ was found to be approximately 365 nm/min, 243 nm/min, and 81 nm/min, respectively. We observed that the etching rate is significantly lower compared to the etching rate presented for AlN (1500 nm/min) in [20]. Like other group III-V materials, ${\mathrm{Sc}}_{x}{\mathrm{Al}}_{1-x}N$ is expected to form oxides compound while reacting with TMAH [$N{\left({\mathrm{CH}}_{3}\right)}_{4}{}^{+}{\mathrm{OH}}^{-}$]. When ${\mathrm{Sc}}_{x}{\mathrm{Al}}_{1-x}N$ and the TMAH solution interact, AlN and ScN form separately and further react to form oxides and amphoteric substance. The following chemical reactions are subjected to happen during the wet etch process:

(1) $$2\mathrm{AlN}+3H_{2}O\frac{N{\left({\mathrm{CH}}_{3}\right)}_{4}{}^{+}{\mathrm{OH}}^{-}}{\to }{\mathrm{Al}}_{2}O_{3}+2{\mathrm{NH}}_{3}$$

(2) $$\mathrm{AlN}+3H_{2}O\frac{N{\left({\mathrm{CH}}_{3}\right)}_{4}{}^{+}{\mathrm{OH}}^{-}}{\to }\mathrm{Al}{\left(\mathrm{OH}\right)}_{3}+{\mathrm{NH}}_{3}$$

(3) $$2\mathrm{ScN}+3H_{2}O\frac{N{\left({\mathrm{CH}}_{3}\right)}_{4}{}^{+}{\mathrm{OH}}^{-}}{\to }{\mathrm{Sc}}_{2}O_{3}+2{\mathrm{NH}}_{3}$$

(4) $$\mathrm{ScN}+3H_{2}O\frac{N{\left({\mathrm{CH}}_{3}\right)}_{4}{}^{+}{\mathrm{OH}}^{-}}{\to }\mathrm{Sc}{\left(\mathrm{OH}\right)}_{3}+{\mathrm{NH}}_{3}$$

The formation of the scandium hydroxide residues occurs at a temperature of <85 °C, and it slows down the etching process of ${\mathrm{Sc}}_{x}{\mathrm{Al}}_{1-x}N$. During the chemical reaction process, other components such as ${\mathrm{CH}}_{4}$, NO and ${\mathrm{NO}}_{2}$ can be formed as well, usually at higher temperatures (>300 °C). Thus, we kept the temperature of the TMAH solution at 80 °C during the etching process to have control of the etching rates of ${\mathrm{Sc}}_{x}{\mathrm{Al}}_{1-x}N$. Finally, the hydroxides that eventually remain on the sample,) can be removed using HF solution with DI water.

## 3. Results and Discussion

**Figure 3:**
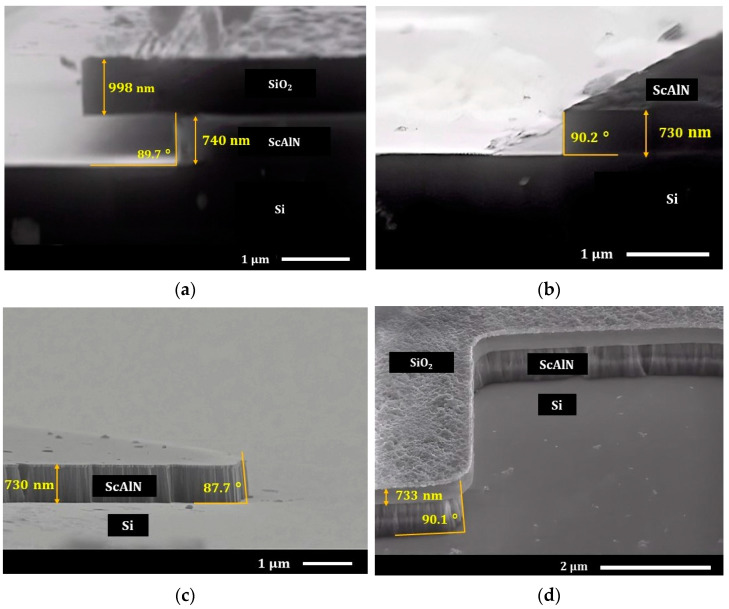
Scanning electron microscope (SEM) images of (**a**) ${\mathrm{Sc}}_{0.20}{\mathrm{Al}}_{0.80}N\;$after 3 min of TMAH etching at 80 °C, (**b**) ${\mathrm{Sc}}_{0.20}{\mathrm{Al}}_{0.80}N\;$ after hard mask (${\mathrm{SiO}}_{2}$) removal, (**c**) ${\mathrm{Sc}}_{0.20}{\mathrm{Al}}_{0.80}N$ after hard mask (SiO_2_) removal with different feature, and (**d**) ${\mathrm{Sc}}_{0.20}{\mathrm{Al}}_{0.80}N$ after TMAH etching with the sample prepared in negative tone (Inverted mask).

**Figure 4:**
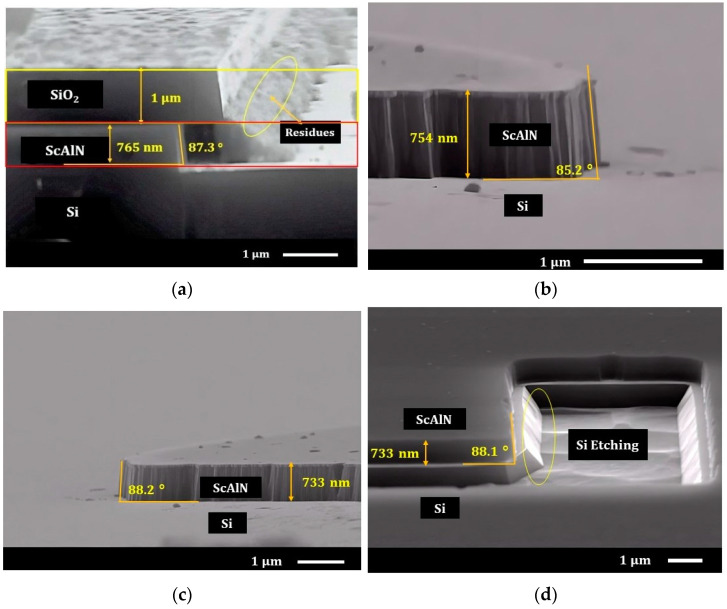
Scanning Electron Microscope (SEM) Images of (**a**) ${\mathrm{Sc}}_{0.40}{\mathrm{Al}}_{0.60}N\;$after 9 min of TMAH Etching at 80 °C, (**b**) ${\mathrm{Sc}}_{0.40}{\mathrm{Al}}_{0.60}N\;$ after Hard Mask (SiO_2_) Removal, (**c**) ${\mathrm{Sc}}_{0.125}{\mathrm{Al}}_{0.875}N\;$ after 2 min of TMAH Etching and Hard Mask (${\mathrm{SiO}}_{2}$) Removal, (**d**) ${\mathrm{Sc}}_{0.20}{\mathrm{Al}}_{0.80}N$ Over etching condition (Silicon undercut).

During the wet etch process, we heated the 25% TMAH solution to 80 °C and then immersed the ${\mathrm{Sc}}_{x}{\mathrm{Al}}_{1-x}N$ samples in the solution. Figure 3a–d shows the wet etch results of the ${\mathrm{Sc}}_{0.20}{\mathrm{Al}}_{0.80}N$ film after 3 min of etching. The 730-nm thick layer was removed in 3 min, resulting in an etch rate of 243 nm/min with 87°~90° vertical sidewalls. This result demonstrates that wet etching using TMAH can be used as an alternative to achieve sidewall angles better than 80°, which can be considered state-of-the-art compared to studies in Table 2. Figure 3c,d illustrates that the same quality of etched profiles can be obtained utilizing different patterns as well as an inverted mask. ${\mathrm{For}\;\mathrm{Sc}}_{0.40}{\mathrm{Al}}_{0.60}N$ ${\mathrm{and}\;\mathrm{Sc}}_{0.125}{\mathrm{Al}}_{0.875}N$, identical TMAH concentration and temperature were used, and the results are shown in Figure 4a–d. The etching rate is found to be relatively lower for ${\mathrm{Sc}}_{0.40}{\mathrm{Al}}_{0.60}N$ (~80 nm/min) compared to ${\mathrm{Sc}}_{0.125}{\mathrm{Al}}_{0.875}N\;$(350 nm/min). This result is consistent with other work, as shown in Table 1 and Table 2. For the lower concentration, the verticality resulted to be 88.2° ± 0.2° (Figure 4d), which is practically the same for what we found for the ${\mathrm{Sc}}_{0.20}{\mathrm{Al}}_{0.80}N$. However, as shown in Figure 4b, the profile of the ${\mathrm{Sc}}_{0.40}{\mathrm{Al}}_{0.60}N$ shows a verticality of ~85° from the provided SEM image.

As the scandium concentration increases, usually the undercut (lateral) etching increases [20,21]. Chen et al., demonstrated the reason behind the dependency of lateral etch with the AOGs [35]. The experiment demonstrated that AOGs are grains that contain ScAlN unit cells that have their c-axis tilted from the normal direction of the film surface and do not precisely nucleate from the bottom of the film [34]. The ${\mathrm{SiO}}_{2}\;$mask is resistant to the etching process and the ${\mathrm{Sc}}_{x}{\mathrm{Al}}_{1-x}N$ film is etched laterally. The result is a suspended ${\mathrm{SiO}}_{2}\;$layer. Thus, we performed high-temperature annealing (thermal diffusion) in the nitrogen atmosphere to recover the surface damage by minimizing the effect of ion bombardment. Annealing is expected to increase the piezoelectric properties of the samples as well [20,36,37,38]. In addition to this, AOGs generated during the growth process increases etch resistivity, especially in wet etch processes [20,39]. In our works, we were successful in reducing the lateral etching with almost vertical sidewalls for higher Sc concentrations (See Figure 4a). This was possible thanks to the combination of the optimized annealing process (temperature and nitrogen concentration) and wet etching recipes (TMAH concentration, temperature, etch time).

One critical observation of this wet etching process is that when the ${\mathrm{Sc}}_{x}{\mathrm{Al}}_{1-x}N\;$sample is withdrawn from the TMAH solution, the low solubility of scandium in alkaline solutions caused the formation of residues. Considering the TMAH chemistry and the work conducted in [20], it is very likely to be that the residues were in the form of ${\mathrm{ScO}}_{x}H_{y}\;$on the sample surface [20], which appeared in the form of morphological substance or bumps (Figure 3a,b,d and Figure 4a). As the scandium content increases, the more significant number of residues will be deposited (See Figure 3a and Figure 4a) that lead to a slower etch. Airola et al. mentioned that there is a dependency of the residues on the rinsing method and etching process [20]; and we have also found that continuously stirring the ${\mathrm{Sc}}_{x}{\mathrm{Al}}_{1-x}N\;$sample during the etching process in TMAH reduces the formation of residues. Therefore, after the TMAH etching process, an effective DI water rinsing is recommended to remove the residues or TMAH will continue to keep the reaction ongoing. Although it will not remove the residues completely in this process, an HF-based cleaning (rinsing) process is highly recommended. Before using HF, we soaked the sample in DI water for 15 min. One of the major reasons behind this process is that if TMAH content remains in the samples during HF cleaning, there is a possibility of generating tetramethylammonium (TMA) salts through the reaction shown in (5).

(5) $$N{\left({\mathrm{CH}}_{3}\right)}_{4}{}^{+}{\mathrm{OH}}^{-}+\;\mathrm{HF}\to {\left({\mathrm{CH}}_{3}\right)}_{4}{}^{+}F^{-}+H_{2}O$$

We used diluted HF solution (3~5%) to remove the hard mask layer (${\mathrm{SiO}}_{2}$). As shown in Figure 3b,c and Figure 4b,c, HF does not influence the verticality or the surface roughness of the ${\mathrm{Sc}}_{x}{\mathrm{Al}}_{1-x}N\;$and contributes to the removal of the residues [See Figure 3b,c and Figure 4b–d]. As expected, the TMAH also etches the Si substrate once it etches the ${\mathrm{Sc}}_{x}{\mathrm{Al}}_{1-x}N\;$film and the etching of Si is very aggressive compared to ${\mathrm{Sc}}_{x}{\mathrm{Al}}_{1-x}N\;$(See Figure 4d). This means that it might be possible to use this method to release the Sc_x_Al_1−x_N from the Si substrate if the ${\mathrm{Sc}}_{x}{\mathrm{Al}}_{1-x}N$ thin film is protected (both upper and underneath) by a layer that is resistant to TMAH.

## 4. Conclusions

In this work, we have successfully developed an efficient process that is capable of etching ${\mathrm{Sc}}_{x}{\mathrm{Al}}_{1-x}N\;\left(x=0.125,\;0.20,\;0.40\right)$, ensuring vertical sidewalls 85°~90° (±0.2°) as well as reducing the degree of undercut. ${\mathrm{Sc}}_{x}{\mathrm{Al}}_{1-x}N$ etching results were found to be independent of the SiO_2_ hard mask thickness, based on the two thicknesses used of 330 nm and 1 μm. We have also analyzed the prospective reasons behind the factors that affect verticality during etching and demonstrated how the annealing process significantly improves the surface damage introduced into the ${\mathrm{Sc}}_{x}{\mathrm{Al}}_{1-x}N\;$by reducing the ion-bombardment effect, which ultimately prevents lateral etching. The more the scandium content increases, the more difficult it is to etch due to the formation of ${\mathrm{ScO}}_{x}H_{y}$ residues with AOG potentially further slowing the wet etch rate. Additionally, the impact of high-temperature annealing can be an additional variable to tune for further optimization of the etching profile. Nevertheless, the reported procedure can yield vertical sidewalls in ${\mathrm{Sc}}_{x}{\mathrm{Al}}_{1-x}N$, which is helpful in the fabrication of piezoelectric devices using Sc_x_Al_1−x_N that are sensitive to sidewall angles.

## Figures and Tables

**Table 1:** Summary ${\mathrm{Sc}}_{x}{\mathrm{Al}}_{1-x}N$ dry etching process results.

Ref.	Material	Etch Process	Etchant Flow (sccm)	ICP/RIE Power (W)	RF Power/Voltage	Pressure (m Torr)	Etch Rate (nm/min)	Mask Used	Sidewall Profile (°)
[23]	${\mathrm{Sc}}_{0.06}{\mathrm{Al}}_{0.94}N$	ICP	${\mathrm{Cl}}_{2}$/${\mathrm{BCl}}_{3}$/$N_{2}$ (15/30/5)	550	80 W	3.8	<230	PR	>77
[24]	${\mathrm{Sc}}_{0.20}{\mathrm{Al}}_{0.80}N$	IBE	Ar 100%	715 V (Beam), 485 mA (Beam)			17	PR	79
		RIBE	Ar/RG (60%/40%)				19		76
		IBE	Ar 100%	900 V (Beam), 600 mA (Beam)			24		71
		RIBE	Ar/RG (75%/25%)				36		73
[18]	${\mathrm{Sc}}_{0.20}{\mathrm{Al}}_{0.8}N$	RIE	${\mathrm{Cl}}_{2}/{\mathrm{BCl}}_{3}/\mathrm{He}$ (90/30/100)	550	150 W	-	160	PR	-
[25]	${\mathrm{Sc}}_{x}{\mathrm{Al}}_{1-x}N$ x = 0.2~0.16	ICP	${\mathrm{Cl}}_{2}/{\mathrm{BCl}}_{3}/$Ar (20/10/10)	200	50 W/(30~70) W	-	3.4	-	-
[26]	${\mathrm{Sc}}_{0.22}{\mathrm{Al}}_{0.78}N$	ICP	${\mathrm{Cl}}_{2}/{\mathrm{BCl}}_{3}/N_{2}$ (25/30/20)	550	350 W	37	130	${\mathrm{SiO}}_{2}$	77

**Table 2:** Summary of ${\mathrm{Sc}}_{x}{\mathrm{Al}}_{1-x}N$ wet etching process results.

Ref.	Material	Etchant	Concentration (%)	Temp (°C)	Etch Rate (nm/min)	Mask	Sidewall (°)
[18]	${\mathrm{Sc}}_{0.15}{\mathrm{Al}}_{0.85}N$	MIF 319 (TMAH)	2.2	60–70	50	-	-
[28]	${\mathrm{Sc}}_{0.20}{\mathrm{Al}}_{0.8}N$	KOH	25	25/40	143	-	-
[20]	${\mathrm{Sc}}_{0.20}{\mathrm{Al}}_{0.8}N$	TMAH	25	80	30–50 (Annealed Sample)	Mo	~80
					40–60 (Annealed Sample)	${\mathrm{SiO}}_{2}/{\mathrm{SiN}}_{x}$	~75
[21]	${\mathrm{Sc}}_{0.125}{\mathrm{Al}}_{0.875}N$	KOH	10	65	80	${\mathrm{SiN}}_{x}$	~92

## Data Availability

The data reported in this manuscript are available on request from the corresponding author.

## Supplementary Materials

The following supporting information can be downloaded at: https://www.mdpi.com/article/10.3390/nano13020274/s1, Figure S1: SEM Image for Sc0.125Al0.875N; Figure S2: EDS Spectrum for Sc0.125Al0.875N; Figure S3: Curve Fitted EDS Spectrum for Sc0.125Al0.875N; Figure S4: SEM Image for Sc0.125Al0.875N; Figure S5: EDS Spectrum for Sc0.125Al0.875N; Figure S6: Curve Fitted EDS Spectrum for Sc0.125Al0.875N; Figure S7: SEM Image for Sc0.125Al0.875N; Figure S8: EDS Spectrum for Sc0.125Al0.875N; Figure S9: Curve Fitted EDS Spectrum for Sc0.125Al0.875N; Figure S10: SEM Image for Sc0.20Al0.80N; Figure S11: EDS Spectrum for Sc0.20Al0.80N; Figure S12: Curve Fitted EDS Spectrum for Sc0.20Al0.80N; Figure S13: SEM Image for Sc0.20Al0.80N; Figure S14: EDS Spectrum for Sc0.20Al0.80N; Figure S15: Curve Fitted EDS Spectrum for Sc0.20Al0.80N; Figure S16: SEM Image for Sc0.20Al0.80N; Figure S17: EDS Spectrum for Sc0.20Al0.80N; Figure S18: Curve Fitted EDS Spectrum for Sc0.20Al0.80N; Figure S19: SEM Image for Sc0.40Al0.60N; Figure S20: EDS Spectrum for Sc0.40Al0.60N; Figure S21: Curve Fitted EDS Spectrum for Sc0.40Al0.60N; Figure S22: SEM Image for Sc0.40Al0.60N; Figure S23: EDS Spectrum for Sc0.40Al0.60N; Figure S24: Curve Fitted EDS Spectrum for Sc0.40Al0.60N; Figure S25: SEM Image for Sc0.40Al0.60N; Figure S26: EDS Spectrum for Sc0.40Al0.60N; Figure S27: Curve Fitted EDS Spectrum for Sc0.40Al0.60N; Figure S28: Curve Fitted EDS Spectrum for Sc0.125Al0.875N, Sc0.20Al0.80N, and Sc0.40Al0.60N; Figure S29: SEM images of Sc0.20Al0.80N sample after TMAH etching (Without Annealing); Figure S30: SEM images of Sc0.20Al0.80N sample After Si02 Etching and High Temper-ature Annealing; Table S1: Quantized EDS Data of Sc0.125Al0.875N (From SEM); Table S2: Quantized EDS Analysis of Sc0.125Al0.875N (Without Nitrogen) (From SEM); Table S3: Quantized EDS Data of Sc0.125Al0.875N (from SEM); Table S4: Quantized EDS Analysis of Sc0.125Al0.875N (Without Nitrogen) (From SEM); Table S5: Quantized EDS Data of Sc0.125Al0.875N (from SEM); Table S6: Quantized EDS Data of Sc0.125Al0.875N (Without Nitrogen) (from SEM); Table S7: Quantized EDS Data of Sc0.20Al0.80N (from SEM); Table S8: Quantized EDS Analysis of Sc0.20Al0.80N (Without Nitrogen) (From SEM); Table S9: Quantized EDS Data of Sc0.20Al0.80N (from SEM); Table S10: Quantized EDS Data of Sc0.20Al0.80N (Without Nitrogen) (from SEM); Table S11: Quantized EDS Data of Sc0.20Al0.80N (from SEM); Table S12: Quantized EDS Data of Sc0.20Al0.80N (Without Nitrogen) (from SEM); Table S13: Quantized EDS Data of Sc0.40Al0.60N (from SEM); Table S14: Quantized EDS Data of Sc0.40Al0.60N (Without Nitrogen) (From SEM); Table S15: Quantized EDS Data of Sc0.40Al0.60N (from SEM); Table S16: Quantized EDS Analysis of Sc0.40Al0.60N (Without Nitrogen) (From SEM); Table S17: Quantized EDS Data of Sc0.40Al0.60N (from SEM); Table S18: Quantized EDS Analysis of Sc0.40Al0.60N (Without Nitrogen) (From SEM).
