# Supplementary material for: Vertical Etching of Scandium Aluminum Nitride Thin Films Using TMAH Solution
Source: Nanomaterials (Basel). 2023 Jan 9;13(2):274. doi: 10.3390/nano13020274 (PMC9863442; doi:10.3390/nano13020274)
Supplement: Supplementary file 1 [file nanomaterials-13-00274-s001.zip › nanomaterials-2085917-supplementary-1.pdf]

Supplementary Materials

Paper Title: Vertical Etching of Scandium Aluminum Nitride Thin Films Using TMAH Solution

A. S. M. Zadid Shifat [1,2,†], Isaac Stricklin [1,3,†], Ravi Kiran Chityala [1,3,†], Arjun Aryal [1,2], Giovanni Esteves [4], Aleem Siddiqui [4], and Tito Busani [1,2,3,*]

[1] Center for High Technology Materials (CHTM), University of New Mexico, Albuquerque, NM, USA.
[2] Optical Science and Engineering (OSE), University of New Mexico, Albuquerque, NM, USA.
[3] Electrical and Computer Engineering (ECE), University of New Mexico, Albuquerque, NM, USA.
[4] Sandia National Laboratories, Albuquerque, NM, USA.

† Authors contributed equally in this work.
* Correspondence: busanit@unm.edu

1. Analysis of $Sc_{0.125}Al_{0.875}N$ Samples

A. $Sc_{0.125}Al_{0.875}N$ (EDS Scan-1)

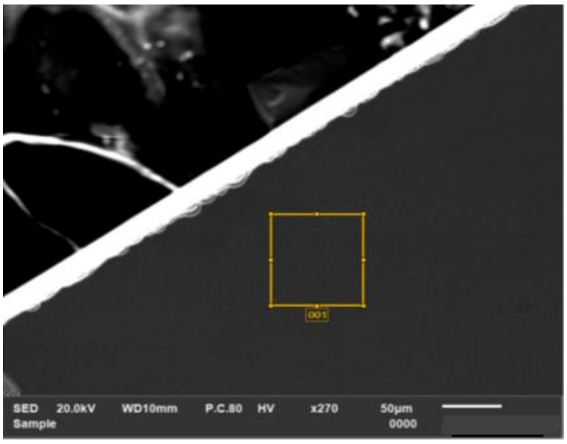

Figure S1: SEM Image for $Sc_{0.125}Al_{0.875}N$

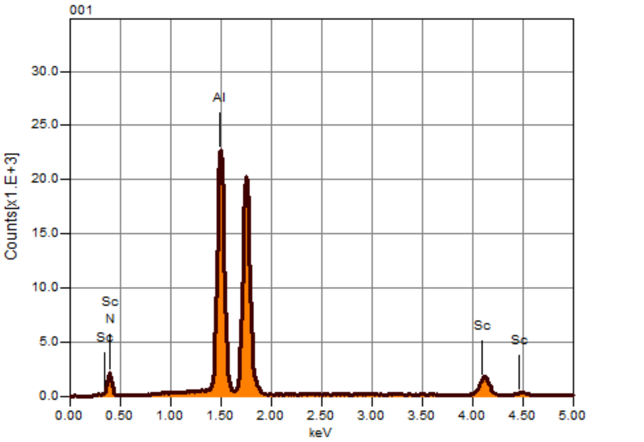

Figure S2: EDS Spectrum for $Sc_{0.125}Al_{0.875}N$

Table S1: Quantized EDS Data of $Sc_{0.125}Al_{0.875}N$ (From SEM)

Formula	mass%	Atom%	Sigma	Net	K Ratio	Line
N*	47.34	65.09	0.09	59367	0.2031719	K
Al	43.31	30.91	0.08	789967	0.2841923	K
Sc*	9.35	4.00	0.05	83052	0.0600187	K
Total	100.00	100.00				

JEOL EDS System JEOL

Table S2: Quantized EDS Analysis of $Sc_{0.125}Al_{0.875}N$ (Without Nitrogen) (From SEM)

Formula	mass%	Atom%	Sigma	Net	K Ratio	Line
Al	80.95	87.57	0.17	789967	0.2841923	K
Sc*	19.04	12.43	0.11	83052	0.0600187	K
Total	100.00	100.00				

JEOL EDS System JEOL

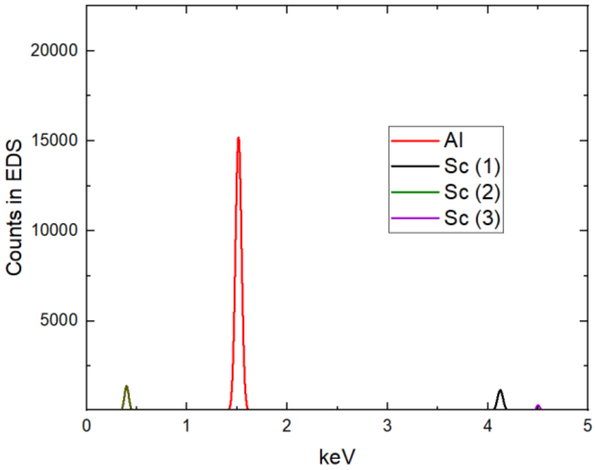

Figure S3: Curve Fitted EDS Spectrum for $Sc_{0.125}Al_{0.875}N$

Calculation:

Based on EDS Analytical Data:

Ratio of Al/Sc Atom% concentration= $\frac{0.8757}{0.1243} = 7.04.$

Area Calculation (EDS Spectrum):

Ratio of Al/Sc Area Spectrum= $\frac{Al}{Sc(1)+Sc(2)+Sc(3)}$

$$= \frac{1414.32}{34.54+171.13+2.42} = 6.81$$

B. $Sc_{0.125}Al_{0.875}N$ (EDS Scan -2)

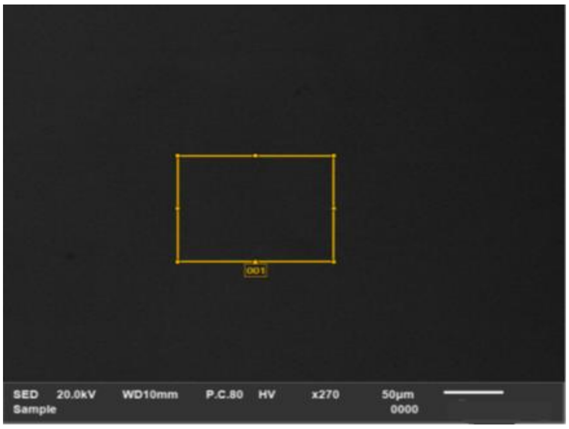

Figure S4: SEM Image for $Sc_{0.125}Al_{0.875}N$

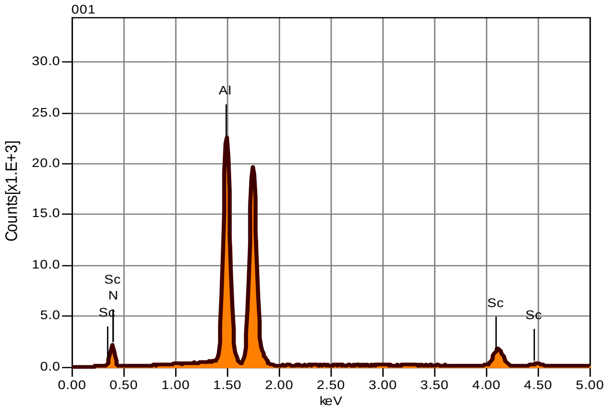

Figure S5: EDS Spectrum for $Sc_{0.125}Al_{0.875}N$

Table S3: Quantized EDS Data of $Sc_{0.125}Al_{0.875}N$ (from SEM)

Formula	mass%	Atom%	Sigma	Net	K ratio	Line
N*	47.69	65.39	0.09	59196	0.2025863	K
Al*	43.06	30.65	0.08	773019	0.2780950	K
Sc*	9.26	3.95	0.05	81067	0.0585846	K
Total	100.00	100.00				

JEOL EDS System

Table S4: Quantized EDS Analysis of $Sc_{0.125}Al_{0.875}N$ (Without Nitrogen) (From SEM)

Formula	mass%	Atom%	Sigma	Net	K Ratio	Line
Al*	79.93	86.90	0.16	773019	0.2780950	K
Sc*	20.07	13.10	0.11	81067	0.0585846	K
Total	100.00	100.00				

JEOL EDS System

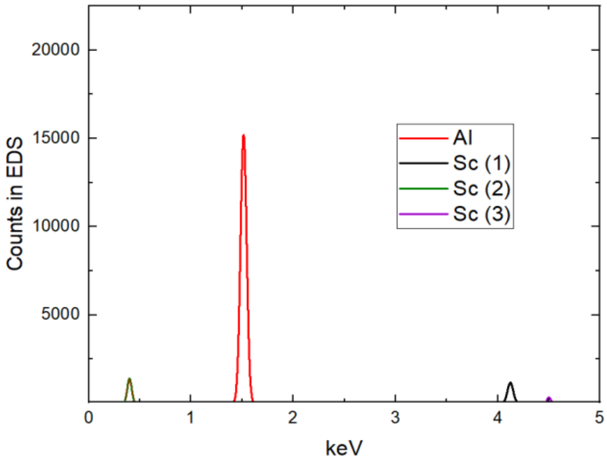

Figure S6: Curve Fitted EDS Spectrum for $Sc_{0.125}Al_{0.875}N$

Calculation:

Based on EDS Analytical Data:

Ratio of Al/Sc Atom% concentration$=\frac{0.8690}{0.1310} = 6.63$

Area Calculation (EDS Spectrum):

$$\text{Ratio of Al/Sc Area Spectrum} = \frac{\text{Al}}{\text{Sc(1)+Sc(2)+Sc(3)}}$$

$$= \frac{1444.31}{37.52+170.43+2.44} = 6.87$$

C. $Sc_{0.125}Al_{0.875}N$ (EDS Scan-3)

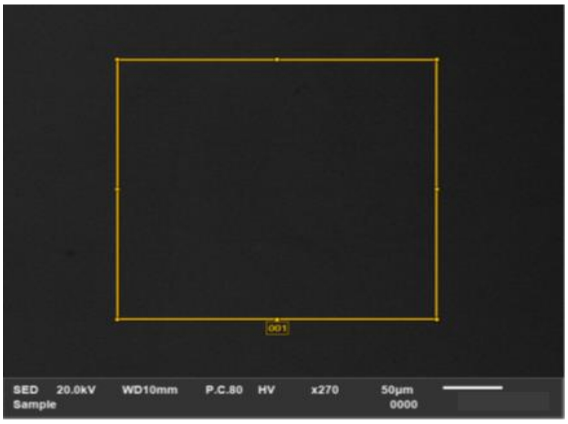

Figure S7: SEM Image for $Sc_{0.125}Al_{0.875}N$

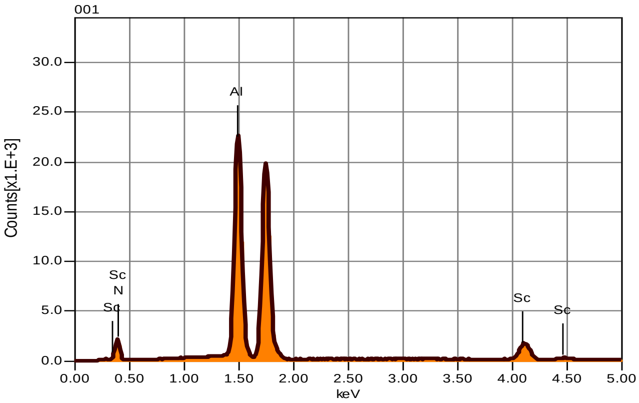

Figure S8: EDS Spectrum for $Sc_{0.125}Al_{0.875}N$

Table S5: Quantized EDS Data of $Sc_{0.125}Al_{0.875}N$ (from SEM)

Formula	mass%	Atom%	Sigma	Net	K Ratio	Line
N*	47.95	65.62	0.09	60108	0.2057052	K
Al*	42.90	30.48	0.08	775150	0.2788618	K
Sc*	9.15	3.90	0.05	80677	0.0583023	K
Total	100.00	100.00				

JEOL EDS System JEOL

Table S6: Quantized EDS Data of $Sc_{0.125}Al_{0.875}N$ (Without Nitrogen) (from SEM)

Formula	mass%	Atom%	Sigma	Net	K Ratio	Line
Al*	80.94	87.55	0.16	775150	0.2788618	K
Sc*	19.06	12.45	0.11	80677	0.0583023	K
Total	100.00	100.00				

JEOL EDS System JEOL

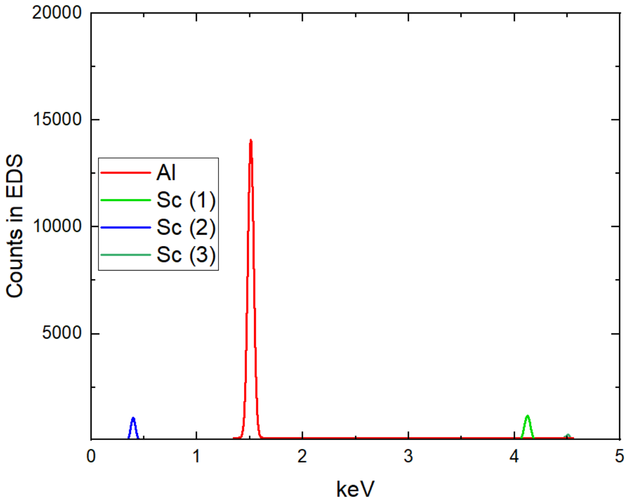

Figure S9: Curve Fitted EDS Spectrum for $Sc_{0.125}Al_{0.875}N$

Calculation:

Based on EDS Analytical Data:

Ratio of Al/Sc Atom% concentration= $\frac{0.8755}{0.1245} = 7.03$

Area Calculation (EDS Spectrum):

Ratio of Al/Sc Area Spectrum= $\frac{Al}{Sc(1)+Sc(2)+Sc(3)}$

$= \frac{1485.62}{34.53+175.33+2.39} = 7.10$

D. Calculation for $Sc_{0.125}Al_{0.875}N$ Sample:

Al/Sc concentration Ratio Average (From EDS Analytical Data) = $\frac{7.04+6.63+7.03}{3} = 6.9$

So, approximate Al/Sc concentration= 87.35% / 12.65%

Al/Sc concentration Ratio Average (From EDS Spectrum Analysis) = $\frac{6.81+6.87+7.10}{3} = 6.93$

So, approximate Al/Sc concentration= 87.39% / 12.61%

2. Analysis of $Sc_{0.20}Al_{0.80}N$ Samples

A. $Sc_{0.20}Al_{0.80}N$ (EDS Scan-1)

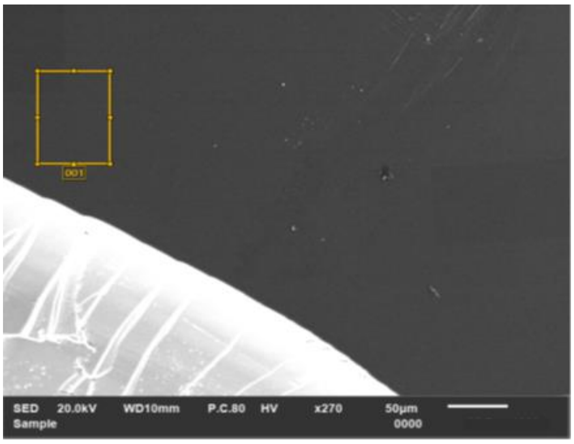

Figure S10: SEM Image for $Sc_{0.20}Al_{0.80}N$

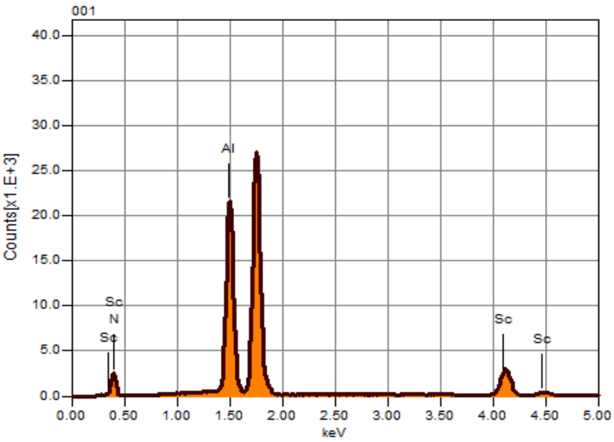

Figure S11: EDS Spectrum for $Sc_{0.20}Al_{0.80}N$

Table S7: Quantized EDS Data of $Sc_{0.20}Al_{0.80}N$ (from SEM)

Formula	mass%	Atom%	Sigma	Net	K ratio	Line
N	47.84	66.46	0.16	19541	0.2526723	K
Al	38.06	27.44	0.14	197636	0.2686397	K
Sc	14.10	6.10	0.10	37125	0.1013702	K
Total	100.00	100.00				

JEOL EDS System JEOL

Table S8: Quantized EDS Analysis of $Sc_{0.20}Al_{0.80}N$ (Without Nitrogen) (From SEM)

Formula	mass%	Atom%	Sigma	Net	K ratio	Line
Al*	71.94	80.94	0.18	385479	0.2623141	K
Sc*	28.06	19.06	0.11	71728	0.0980495	K
Total	100.00	100.00				

JEOL EDS System JEOL

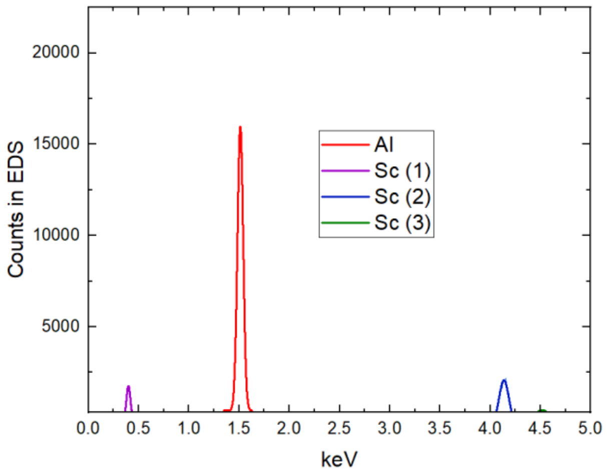

Figure S12: Curve Fitted EDS Spectrum for $Sc_{0.20}Al_{0.80}N$

Calculation:

Based on EDS Analytical Data:

Ratio of Al/Sc Atom% concentration$=\frac{0.8094}{0.1906}=4.24$

Area Calculation (EDS Spectrum):

Ratio of Al/Sc Area Spectrum$=\frac{Al}{Sc(1)+Sc(2)+Sc(3)}$

$$=\frac{1217.45}{217.76+76.92+3.19}=4.13$$

B. $Sc_{0.20}Al_{0.80}N$ (EDS Scan-2)

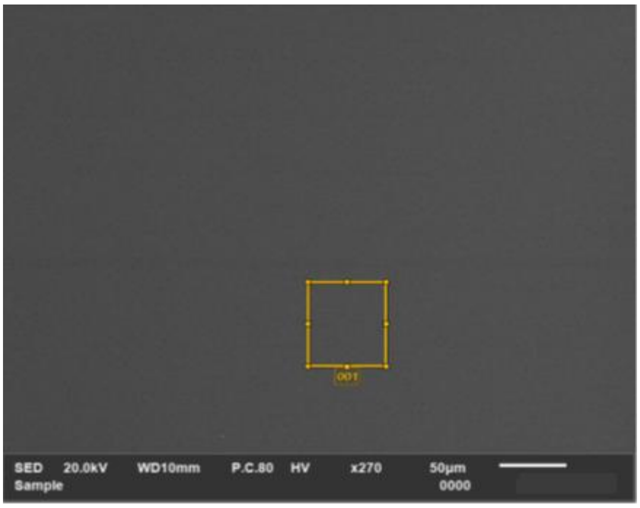

Figure S13: SEM Image for $Sc_{0.20}Al_{0.80}N$

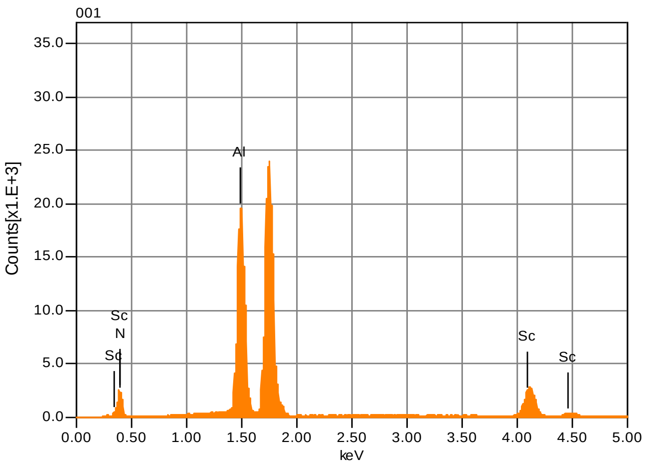

Figure S14: EDS Spectrum for $Sc_{0.20}Al_{0.80}N$

Table S9: Quantized EDS Data of $Sc_{0.20}Al_{0.80}N$ (from SEM)

Formula	mass%	Atom%	Sigma	Net	K ratio	Line
N	47.52	66.20	0.10	45068	0.2278225	K
Al	38.12	27.56	0.09	460000	0.2444402	K
Sc	14.36	6.23	0.07	87902	0.0938310	K
Total	100.00	100.00				

JEOL EDS System JEOL

Table S10: Quantized EDS Data of $Sc_{0.20}Al_{0.80}N$ (Without Nitrogen) (from SEM)

Formula	mass%	Atom%	Sigma	Net	K ratio	Line
Al	71.28	80.15	0.14	460000	0.2444402	K
Sc	28.72	19.85	0.12	87902	0.0938310	K
Total	100.00	100.00				

JEOL EDS System JEOL

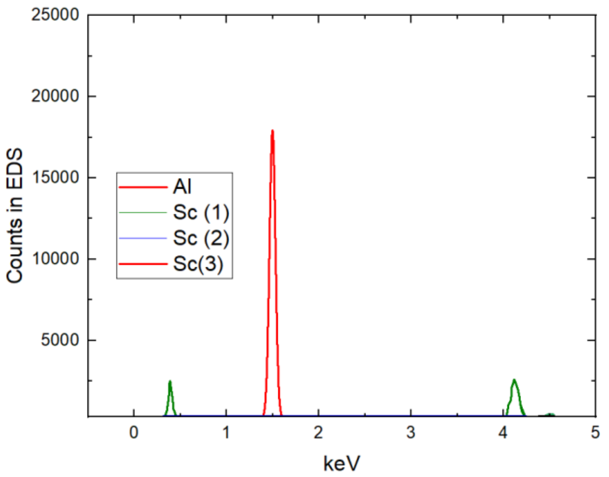

Figure S15: Curve Fitted EDS Spectrum for $Sc_{0.20}Al_{0.80}N$

<u>Calculation</u>:

Based on EDS Analytical Data:

Ratio of Al/Sc Atom% concentration= $\frac{0.8015}{0.1995}$ = 4.012

Area Calculation (EDS Spectrum):

Ratio of Al/Sc Area Spectrum= $\frac{Al}{Sc(1)+Sc(2)+Sc(3)}$

$$= \frac{1321.80}{268.03+51.20+1.19} = 4.11$$

C. $Sc_{0.20}Al_{0.80}N$ (EDS Scan-3)

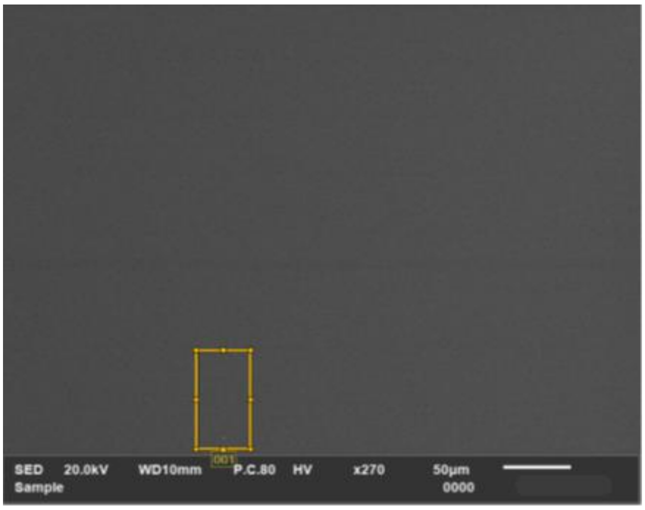

Figure S16: SEM Image for $Sc_{0.20}Al_{0.80}N$

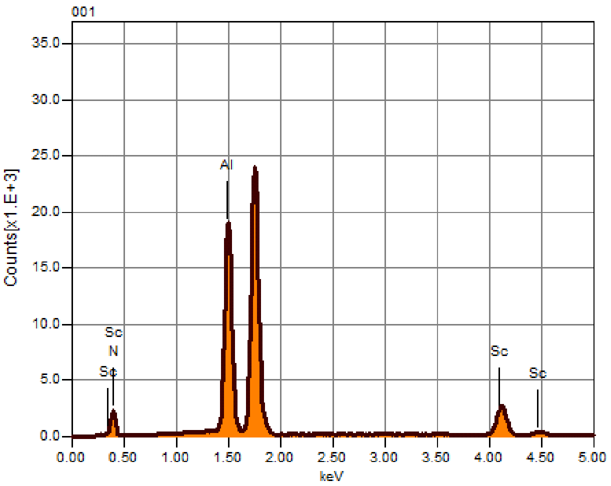

Figure S17: EDS Spectrum for $Sc_{0.20}Al_{0.80}N$

Table S11: Quantized EDS Data of $Sc_{0.20}Al_{0.80}N$ (from SEM)

Formula	mass%	Atom%	Sigma	Net	K Ratio	Line
N	47.71	66.39	0.09	65619	0.2245672	K
Al	37.89	27.37	0.08	659629	0.2373030	K
Sc	14.40	6.24	0.06	127302	0.0919972	K
Total	100.00	100.00				

JEOL EDS System

Table S12: Quantized EDS Data of $Sc_{0.20}Al_{0.80}N$ (Without Nitrogen) (from SEM)

Formula	mass%	Atom%	Sigma	Net	K Ratio	Line
Al	71.20	80.06	0.08	659629	0.2373030	K
Sc	28.80	19.94	0.06	127302	0.0919972	K
Total	100.00	100.00				

JEOL EDS System

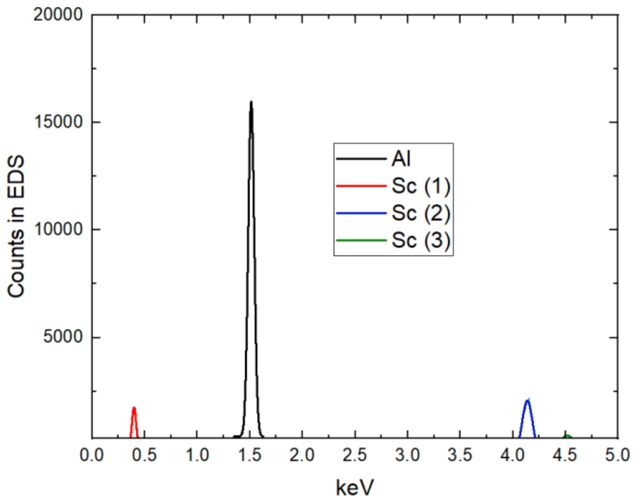

Figure S18: Curve Fitted EDS Spectrum for $Sc_{0.20}Al_{0.80}N$

<u>Calculation</u>:

Based on EDS Analytical Data:

Ratio of Al/Sc Atom% concentration= $\frac{0.8015}{0.1995}$ = 4.02

Area Calculation (EDS Spectrum):

Ratio of Al/Sc Area Spectrum= $\frac{Al}{Sc(1)+Sc(2)+Sc(3)}$

$= \frac{1318.60}{261.53+58.20+1.23} = 4.11$

<u>D. Concentration Calculation for $Sc_{0.20}Al_{0.80}N$ Sample:</u>

Al/Sc concentration Ratio Average (From EDS Analytical Data) = $\frac{4.24+4.012+4.02}{3} = 4.09$

So, approximate Al/Sc concentration= 80.35% / 19.65%

Al/Sc concentration Ratio Average (From EDS Spectrum Analysis) = $\frac{4.13+4.11+4.13}{3} = 4.12$

So, approximate Al/Sc concentration= 80.47% / 19.53%

<u>3. Analysis of $Sc_{0.40}Al_{0.60}N$ Samples</u>

<u>A. $Sc_{0.40}Al_{0.60}N$ (EDS Scan-1)</u>

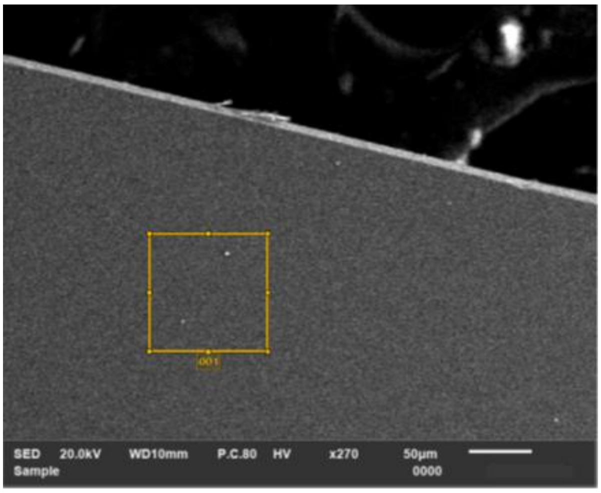

Figure S19: SEM Image for $Sc_{0.40}Al_{0.60}N$

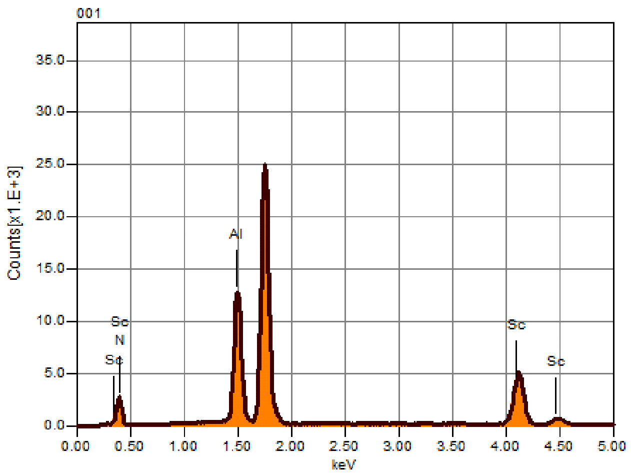

Figure S20: EDS Spectrum for $Sc_{0.40}Al_{0.60}N$

Table S13: Quantized EDS Data of $Sc_{0.40}Al_{0.60}N$ (from SEM)

Formula	mass%	Atom%	Sigma	Net	K Ratio	Line
N*	45.18	66.43	0.08	75196	0.2573417	K
Al*	27.69	21.14	0.07	441265	0.1587460	K
Sc*	27.13	12.43	0.07	238052	0.1720323	K
Total	100.00	100.00				

JEOL EDS System JEOL

Table S14: Quantized EDS Data of $Sc_{0.40}Al_{0.60}N$ (Without Nitrogen) (From SEM)

Formula	mass%	Atom%	Sigma	Net	K Ratio	Line
Al*	51.07	61.98	0.11	441265	0.1587460	K
Sc*	48.93	38.02	0.11	238052	0.1720323	K
Total	100.00	100.00				

JEOL EDS System JEOL

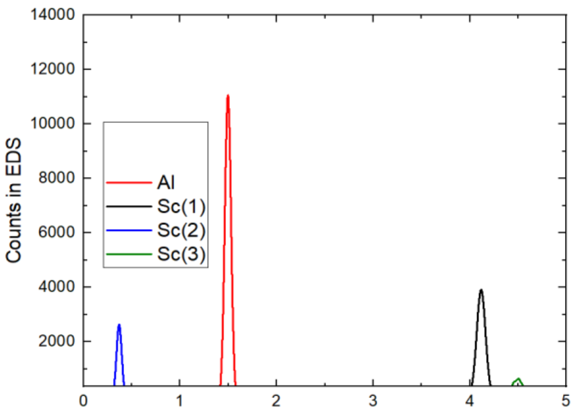

Figure S21: Curve Fitted EDS Spectrum for $Sc_{0.40}Al_{0.60}N$

<u>Calculation</u>:

Based on EDS Analytical Data:

Ratio of Al/Sc Atom% concentration$=\frac{0.6198}{0.3802}=1.63$

Area Calculation (EDS Spectrum):

Ratio of Al/Sc Area Spectrum$=\frac{\mathrm{Al}}{\mathrm{Sc(1)+Sc(2)+Sc(3)}}$

$$=\frac{919.09}{432.61+171.52+3.49}=1.51$$

<u>B. $Sc_{0.40}Al_{0.60}N$ (EDS Scan-2)</u>

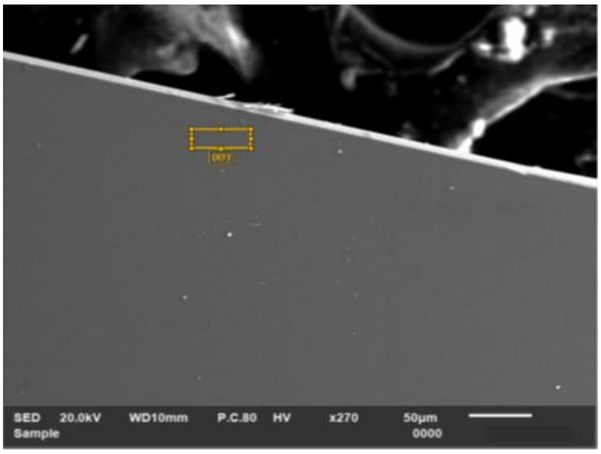

Figure S22: SEM Image for $Sc_{0.40}Al_{0.60}N$

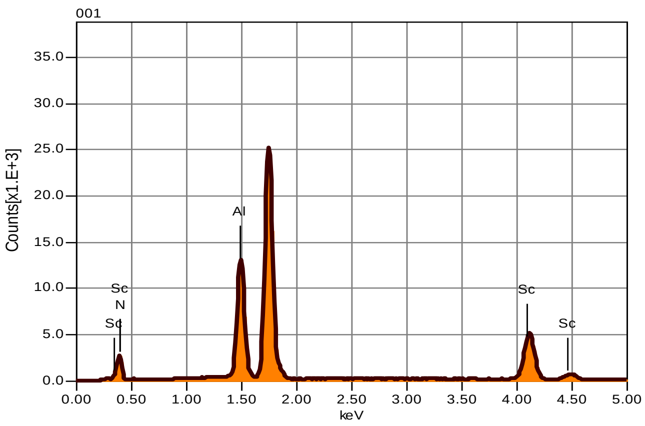

Figure S23: EDS Spectrum for $Sc_{0.40}Al_{0.60}N$

Table S15: Quantized EDS Data of $Sc_{0.40}Al_{0.60}N$ (from SEM)

Formula	mass%	Atom%	Sigma	Net	K Ratio	Line
N	44.98	66.27	0.08	75432	0.2581488	K
Al	27.74	21.22	0.07	445990	0.1604458	K
Sc	27.27	12.52	0.07	241323	0.1743959	K
Total	100.00	100.00				

JEOL EDS System

Table S16: Quantized EDS Analysis of $Sc_{0.40}Al_{0.60}N$ (Without Nitrogen) (From SEM)

Formula	mass%	Atom%	Sigma	Net	K Ratio	Line
Al	52.73	62.46	0.07	445990	0.1604458	K
Sc	47.27	37.54	0.07	241323	0.1743959	K
Total	100.00	100.00				

JEOL EDS System

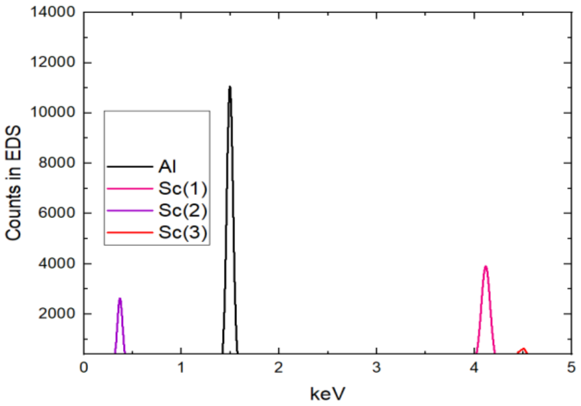

Figure S24: Curve Fitted EDS Spectrum for $Sc_{0.40}Al_{0.60}N$

<u>Calculation</u>:

Based on EDS Analytical Data:

Ratio of Al/Sc Atom% concentration$=\frac{0.6246}{0.3754}=1.65$

Area Calculation (EDS Spectrum):

Ratio of Al/Sc Area Spectrum$=\frac{\mathrm{Al}}{\mathrm{Sc(1)+Sc(2)+Sc(3)}}$

$$=\frac{939.09}{433.31+174.41+3.23}=1.54$$

C. $Sc_{0.40}Al_{0.60}N$ (EDS Scan-3)

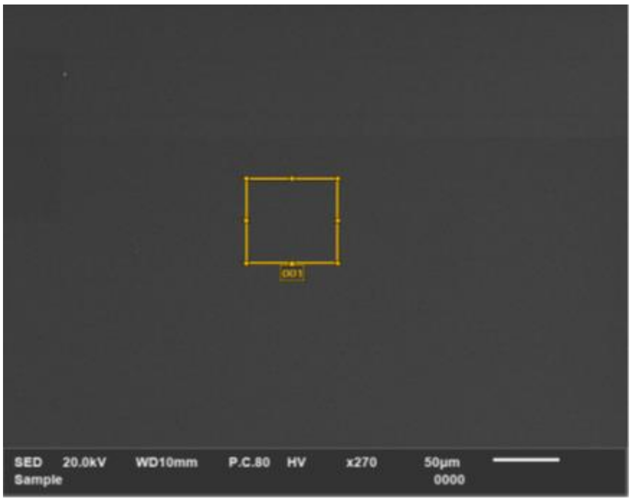

Figure S25: SEM Image for $Sc_{0.40}Al_{0.60}N$

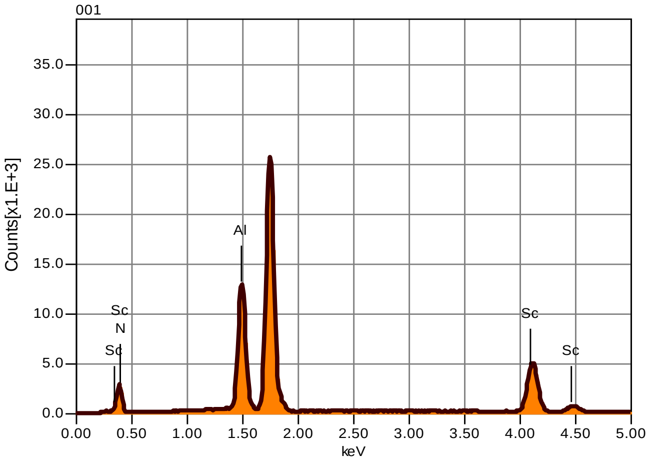

Figure S26: EDS Spectrum for $Sc_{0.40}Al_{0.60}N$

Table S17: Quantized EDS Data of $Sc_{0.40}Al_{0.60}N$ (from SEM)

Formula	mass%	Atom%	Sigma	Net	K Ratio	Line
N	45.18	66.46	0.08	76339	0.2612539	K
Al	27.55	21.04	0.07	444082	0.1597595	K
Sc	27.27	12.50	0.07	242173	0.1750101	K
Total	100.00	100.00				

JEOL EDS System JEOL

Table S18: Quantized EDS Analysis of $Sc_{0.40}Al_{0.60}N$ (Without Nitrogen) (From SEM)

Formula	mass%	Atom%	Sigma	Net	K Ratio	Line
Al	51.73	61.74	0.11	444082	0.1597595	K
Sc	48.27	38.26	0.11	242173	0.1750101	K
Total	100.00	100.00				

JEOL EDS System JEOL

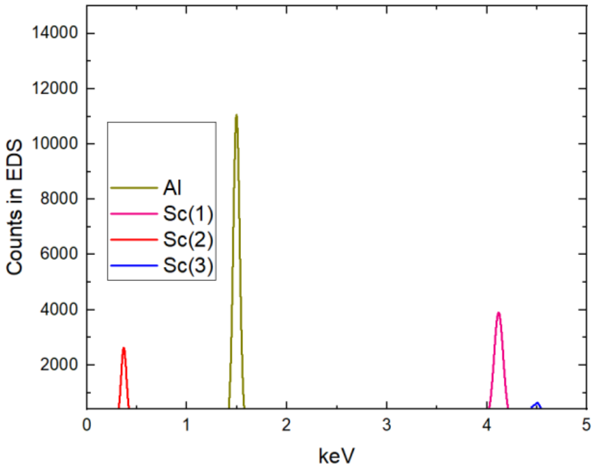

Figure S27: Curve Fitted EDS Spectrum for $Sc_{0.40}Al_{0.60}N$

Calculation:

Based on EDS Analytical Data:

Ratio of Al/Sc Atom% concentration= $\frac{0.6174}{0.3826} = 1.61$

Area Calculation (EDS Spectrum):

Ratio of Al/Sc Area Spectrum= $\frac{Al}{Sc(1)+Sc(2)+Sc(3)}$

$= \frac{931.09}{435.49+169.31+2.91} = 1.53$

D. Concentration Calculation for $Sc_{0.20}Al_{0.80}N$ Sample:

Al/Sc concentration Ratio Average (From EDS Analytical Data) = $\frac{1.61+1.63+1.65}{3} = 1.63$

So, approximate Al/Sc concentration= 61.98% / 38.02%

Al/Sc concentration Ratio Average (From EDS Spectrum Analysis) = $\frac{1.51+1.54+1.53}{3} = 1.53$

So, approximate Al/Sc concentration= 60.48% / 39.52%

4. Scandium Concentration (With Average Data) Analysis:

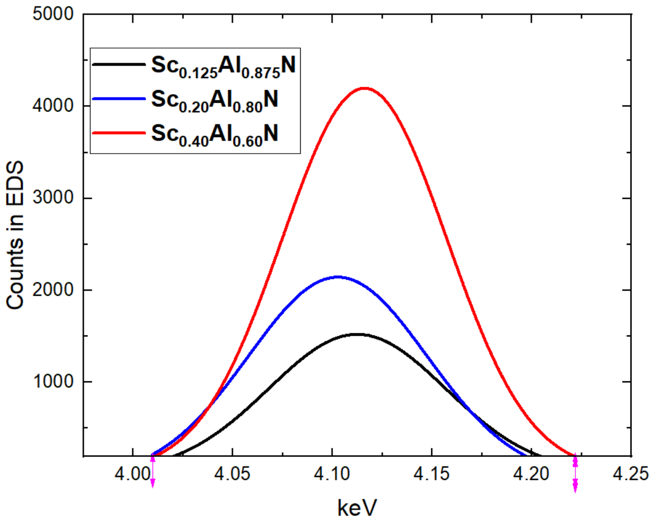

Figure S28: Curve Fitted EDS Spectrum for $Sc_{0.125}Al_{0.875}N$, $Sc_{0.20}Al_{0.80}N$, and $Sc_{0.40}Al_{0.60}N$

Calculation:

Area Ratio of Scandium concentration curve of $Sc_{0.20}Al_{0.80}N$, and $Sc_{0.125}Al_{0.875}N$ sample=

$$\frac{\text{Area for Sc0.20Al0.80N sample}}{\text{Area for Sc0.125Al0.875N sample}} = 1.62 \text{ (Approx)}$$

Area Ratio of Scandium concentration curve of $Sc_{0.40}Al_{0.60}N$, and $Sc_{0.20}Al_{0.80}N$ sample=

$$\frac{\text{Area for Sc0.40Al0.60N sample}}{\text{Area for Sc0.20Al0.80N sample}} = 1.97 \text{ (Approx)}$$

Area Ratio of Scandium concentration curve of $Sc_{0.40}Al_{0.60}N$, and $Sc_{0.125}Al_{0.875}N$ sample=

$$\frac{\text{Area for Sc0.40Al0.60N sample}}{\text{Area for Sc0.125Al0.875N sample}} = 3.17 \text{ (Approx)}$$

5. SEM Images of $Sc_xAl_{1-x}N$ sample in different conditions

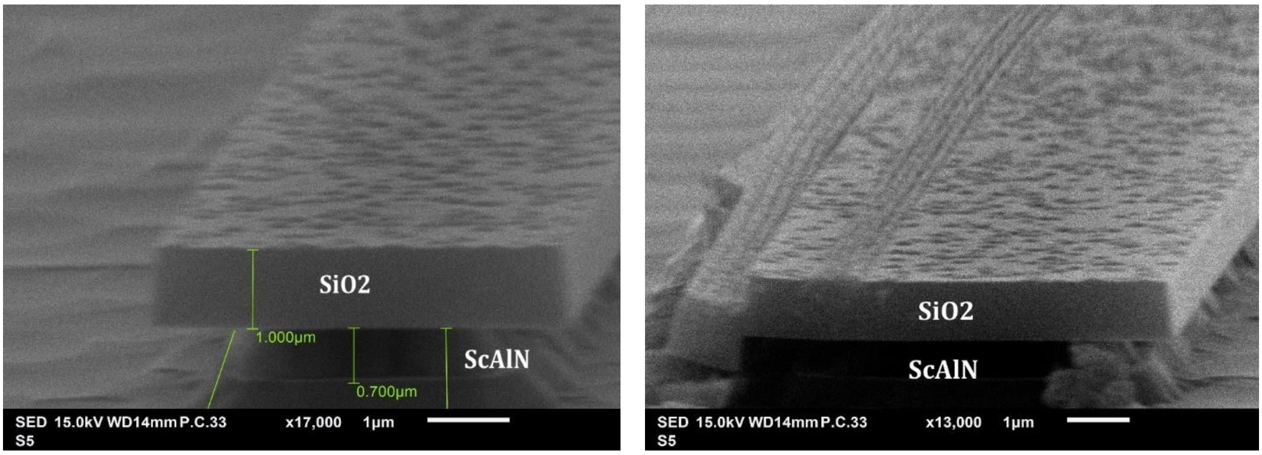

Figure S29: SEM images of $Sc_{0.20}Al_{0.80}N$ sample after TMAH etching (Without Annealing)

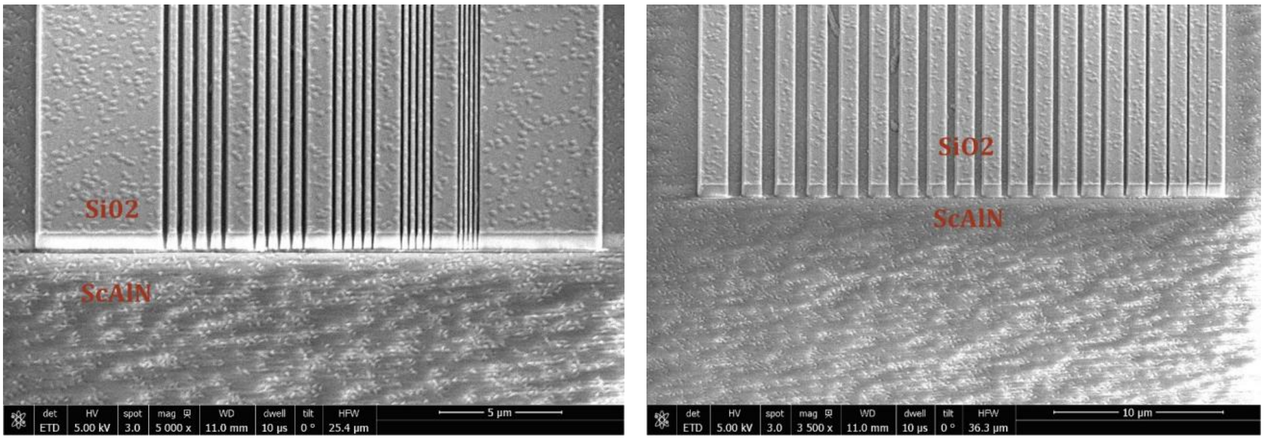

Figure S30: SEM images of $Sc_{0.20}Al_{0.80}N$ sample After SiO_2 Etching and High Temperature Annealing
